# Heterogeneous Multiple Sensors Joint Tracking of Maneuvering Target in Clutter

**DOI:** 10.3390/s150717350

**Published:** 2015-07-17

**Authors:** Panlong Wu, Xingxiu Li, Jianshou Kong, Jiale Liu

**Affiliations:** 1 Department of Automation, Nanjing University of Science and Technology, No.200, Xiaolingwei Street, Xuanwu District, Nanjing 210094, China; E-Mails: plwu@njust.edu.cn (P.W.); ljle126@126.com (J.L.); 2 Department of Science, Nanjing University of Science and Technology, No.200, Xiaolingwei Street, Xuanwu District, Nanjing 210094, China; E-Mail: xxlwpl@126.com

**Keywords:** centralized fusion, debiased converted measurement, interacting multiple model, probability data association, target tracking

## Abstract

To solve the problem of tracking maneuvering airborne targets in the presence of clutter, an improved interacting multiple model probability data association algorithm (IMMPDA-MDCM) using radar/IR sensors fusion is proposed. Under the architecture of the proposed algorithm, the radar/IR centralized fusion tracking scheme of IMMPDA-MDCM is designed to guarantee the observability of the target state. The interacting multiple model (IMM) deals with the model switching. The modified debiased converted measurement (MDCM) filter accounts for non-linearity in the dynamic system models, and reduces the effect of measurement noise on the covariance effectively. The probability data association (PDA) handles data association and measurement uncertainties in clutter. The simulation results show that the proposed algorithm can improve the tracking precision for maneuvering target in clutters, and has higher tracking precision than the traditional IMMPDA based on EKF and IMMPDA based on DCM algorithm.

## 1. Introduction

Target tracking is an essential requirement for the fire control system of the armed reconnaissance vehicle, which is equipped with a suite of advanced sensors to detect, locate, track, classify and automatically identify targets under all climatic conditions. The sensors fusion system has the superiority over the conventional one with a single sensor in target tracking [1]. Active and passive sensors are mutually independent or complementary to target detection and tracking. The radar is an active sensor, which has narrow beam width and high precision of range measurement. However, it is easy to be interfered with by electromagnetic field. The infrared (IR) sensor is a passive system, which is quite sensitive to atmospheric conditions and has no effect on electromagnetic interference. Furthermore, it has higher precision of angular measurements than radar [2]. The radar/IR fusion system could considerably improve the tracking precision by using their complementary characteristics. However, the measurements of the radar and IR sensors are nonlinear and the target is maneuverable. Therefore, the nonlinear filter for maneuvering target tracking should be researched for radar/IR fusion system.

A promising approach to track a maneuvering target is the interacting multiple model (IMM) algorithm. The IMM is built from a finite number of dynamic models that represent different target behavioral traits [3], which makes it natural to track maneuvering target. A converted measurement IMM filter was proposed for tracking a maneuvering target using radar/IR sensors [4]. A new distributed fusion method of radar/IR tracking system based on separation and combination of the measurements was proposed in [5]. A distributed flow of information fusion for radar/IR compound seeker was established in [6], and the federated filter was used to track the target. An adaptive grid IMM based on modified iterated extended Kalman filter for tracking a maneuvering target using radar/ IR sensors was proposed in [7].

In the radar/IR sensor compound tracking system, the tracking of an airborne target in a cluttered environment might be a challenge due to the several observations for a single airborne target, some tracking measurements do not originate from the airborne target. Therefore, the present study utilizes the probabilistic data association (PDA) filter [8,9] to assign weights to the validated measurements. The PDA filter can extend the tracking capability to a highly cluttered environment. Combining IMM with appropriate data association algorithm can realize maneuvering target tracking in clutters [10,11], such as maximum likelihood probabilistic data association (ML-PDA), IMMPDA, interacting multiple model multiple hypothesis tracking (IMM-MHT) and so on. A ML-PDA algorithm has been shown to be robust in a cluttered environment for a constant velocity target, however, it cannot be applied to the situation where targets undergo maneuvers. An adaptive update rate tracking algorithm based on modified IMMPDA is proposed to avoid tracking loss of maneuvering target tracking in clutters [12]. An interacting multiple model probability data association (IMMPDA) algorithm was proposed to support the navigation and surveillance services of the air traffic management system [13]. 

The dynamic of target is usually modeled and tracked in the Cartesian coordinates, whereas the measurements are provided in terms of range and angle with respect to the sensor location in the polar coordinates. Therefore, the radar/IR compound tracking becomes a kind of non-liner estimation problem. One solution to this problem is the extended Kalman filter (EKF), but would results in filter divergence [14,15]. The other solution is debiased converted measurement (DCM) Kalman filter [16], which converts the polar measurements to Cartesian coordinates and then filtering in the Cartesian coordinates. In this paper, the IMMPDA algorithm is combined with the modified debiased converted measurement (MDCM) filter to create an IMMPDA-MDCM filter for an airborne maneuvering target tracking in radar/IR fusion system. The Monte Carlo simulation results show that the proposed IMMPDA-MDCM kalman filter (IMMPDA-MDCMKF) algorithm can improve the target tracking precision, credibility and outperform the conventional algorithms.

The remainder of this paper is organized as follows. In Section 2, the sensor measurement model is derived. In Section 3, the time alignment and fusion of radar and IR sensors are derived. The IMMPDA-MDCM algorithm is proposed in Section 4. In Section 5, the simulation results demonstrate the feasibility and precision of the proposed algorithm. Conclusions are drawn in Section 6.

## 2. The Sensor Measurement Model

**Figure 1:**
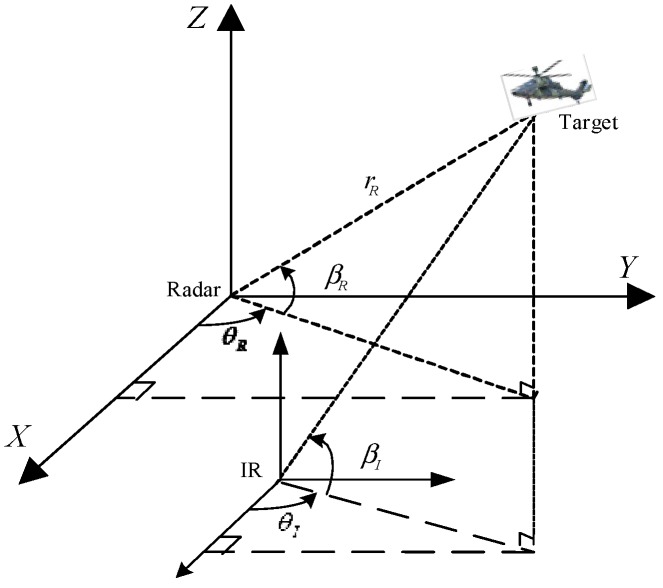
Geometry measuring relationship between target and radar/IR platform.

Considering an arbitrary maneuvering target in 3D Cartesian coordinates, the geometry measuring relationship between target and radar/IR platform is described in Figure 1.

The range $r_{Rk}$, azimuth $\theta_{Rk}$ and elevation $\beta_{Rk}$ can be measured by radar, and the radar measurement is $Z_{Rk}={\left[r_{Rk},\theta_{Rk},\beta_{Rk}\right]}^{T}$ .Let the state vector of target is $X_{k}={\left[x_{k},y_{k},z_{k},{\dot{x}}_{k},{\dot{y}}_{k},{\dot{z}}_{k},{\ddot{x}}_{k},{\ddot{y}}_{k},{\ddot{z}}_{k}\right]}^{T}$. The radar measurement equation is

(1) $$\begin{array}{l} Z_{Rk}=\left[\begin{array}{c} \sqrt{x_{k}^{2}+y_{k}^{2}+z_{k}^{2}}\\ \arctan\frac{y_{k}}{x_{k}}\\ \arcsin\frac{z_{k}}{\sqrt{x_{k}^{2}+y_{k}^{2}+z_{k}^{2}}} \end{array}\right]+\left[\begin{array}{c} v_{r_{R},k}\\ v_{\theta_{R},k}\\ v_{\beta_{R},k} \end{array}\right]\text{=}h_{R}\text{(}X_{k}\text{)+}v_{Rk} \end{array}$$

where $\left(x_{k},y_{k},z_{k}\right)$, $\left({\dot{x}}_{k},{\dot{y}}_{k},{\dot{z}}_{k}\right)$, and $\left({\ddot{x}}_{k},{\ddot{y}}_{k},{\ddot{z}}_{k}\right)$ represent target position, velocity, and acceleration components in *x*, *y*, and *z* direction, respectively. $v_{r_{Rk}}$, $v_{\theta_{Rk}}$ and $v_{\beta_{Rk}}$ are separately independent identically distributed (i.i.d) zero-mean Gaussian white noise, with variance $\sigma_{r_{Rk}}^{2}$, $\sigma_{\theta_{Rk}}^{2}$ and $\sigma_{\beta_{Rk}}^{2}$ respectively. The measurement noise of $Z_{Rk}$ is $\sigma_{Rk}^{2}=diag[\sigma_{r_{Rk}}^{2},\sigma_{\theta_{Rk}}^{2},\sigma_{\beta_{Rk}}^{2}]$.

The measurements of IR sensor is $Z_{Ik}={\left[r_{Ik},\theta_{Ik},\beta_{Ik}\right]}^{T}$, the IR measurement equation is

(2) $$Z_{Ik}=\left[\begin{array}{c} \arctan\frac{y_{k}}{x_{k}}\\ \arcsin\frac{z_{k}}{\sqrt{x_{k}^{2}+y_{k}^{2}+z_{k}^{2}}} \end{array}\right]+\left[\begin{array}{c} v_{\theta_{Ik}}\\ v_{\beta_{Ik}} \end{array}\right]\text{=}h_{I}\text{(}X_{k}\text{)+}v_{Ik}$$

where $v_{\theta_{Ik}}$ and $v_{\beta_{Ik}}$ are separately i.i.d zero-mean Gaussian white noise with variance $\sigma_{\theta_{Ik}}^{2}$ and $\sigma_{\beta_{Ik}}^{2}$ respectively. The measurement noise variance of $Z_{Ik}$ is $\sigma_{Ik}^{2}={\left[\sigma_{\theta_{Ik}}^{2}\sigma_{\beta_{Ik}}^{2}\right]}^{T}$.

## 3. Data Fusion with Radar and IR Sensors

For the convenience of discussion, we assume that radar and IR sensors lie in the same platform. Compared with the detected target, the distance between them is negligible. Therefore, it can be assumed that the two sensors are located in the same position. 

**Figure 2:**
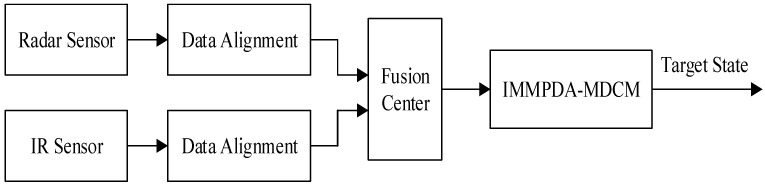
Centralized fusion tracking architecture with radar and IR sensors.

Nowadays, most information fusion algorithms for the compound tracking system are using centralized fusion structure. In this paper, the centralized fusion architecture is used before IMMPDA-MDCM filtering. Because the measurements from radar and IR sensors are independent of each other, each sensor transmits data to the fusion center at different sampling period. Therefore, the measured data from different sensors should be synchronized, and time alignment is needed. The diagram of radar/IR fusion tracking architecture is shown in Figure 2.

### 3.1. Time Alignment of Radar and IR

Suppose that the sampling period of radar and IR sensors are *T_R_* and *T_I_*, respectively, and *T_R_*:*T_I_ = m*:*n*. The two sensors can be synchronized once at *T* (the smallest common multiple of *T_R_* and *T_I_*). Therefore, it is reasonable to select *T* to be the sampling period of tracking system. In time *T*, radar and IR sensors have *n* and *m* samples, respectively. Common algorithms of time alignment include least square method, interpolation, extrapolation and curve fitting algorithm [17]. Because of the good real-time performance, the least square algorithm is chosen in this paper.

(3) $${\hat{Z}}_{Rk}=c_{1}\sum_{i=1}^{n}Z_{Rk}^{i}+c_{2}\sum_{i=1}^{n}iZ_{Rk}^{i}$$

(4) $${\hat{Z}}_{Ik}=d_{1}\sum_{i=1}^{m}Z_{Ik}^{i}+d_{2}\sum_{i=1}^{m}iZ_{Ik}^{i}$$

where $c_{1}=-2/n$, $c_{2}=6/n(n+1)$, $d_{1}=-2/m$, $d_{2}=6/m(m+1)$. After alignment, the measurement noise variances of radar and IR sensors are denoted as

(5) $${\hat{\sigma }}_{Rk}^{2}=\frac{2\sigma_{Rk}^{2}(2n+1)}{n(n+1)}$$

(6) $${\hat{\sigma }}_{Ik}^{2}=\frac{2\sigma_{Ik}^{2}(2m+1)}{m(m+1)}$$

### 3.2. The Fusion of Synchronized Data

In this paper, the weighted average algorithm is used for the fusion of azimuth and elevation measurements. In this fusion algorithm, the constrained extremum calculated through Lagrange multiplier algorithm is chosen as the weighted coefficient, and the fusion precision can approximate the optimal. The fusion measurements and variance of azimuth are denoted as

(7) $$\theta_{k}={\bar{\sigma }}_{\theta k}^{2}\left[\frac{{\hat{\theta }}_{Rk}}{{\hat{\sigma }}_{\theta_{Rk}}^{2}}+\frac{{\hat{\theta }}_{Ik}}{{\hat{\sigma }}_{\theta_{Ik}}^{2}}\right]$$

(8) $${\bar{\sigma }}_{\theta k}^{2}=\frac{{\hat{\sigma }}_{\theta_{Rk}}^{2}{\hat{\sigma }}_{\theta_{Ik}}^{2}}{{\hat{\sigma }}_{\theta_{Rk}}^{2}+{\hat{\sigma }}_{\theta_{Ik}}^{2}}$$

The fusion measurements and variance of elevation are denoted as

(9) $$\beta_{k}={\bar{\sigma }}_{\beta k}^{2}\left[\frac{{\hat{\beta }}_{Rk}}{{\hat{\sigma }}_{\beta_{Rk}}^{2}}+\frac{{\hat{\beta }}_{Ik}}{{\hat{\sigma }}_{\beta_{Ik}}^{2}}\right]$$

(10) $${\bar{\sigma }}_{\beta k}^{2}=\frac{{\hat{\sigma }}_{\beta_{Rk}}^{2}{\hat{\sigma }}_{\beta_{Ik}}^{2}}{{\hat{\sigma }}_{\beta_{Rk}}^{2}+{\hat{\sigma }}_{\beta_{Ik}}^{2}}$$

The time aligned range ${\hat{r}}_{R,k}$ and the fusion angles are merged into an augmented measurement vector as

(11) $$Z_{k}=h(X_{k},v_{k})=\left[\begin{array}{c} {\hat{r}}_{Rk}\\ \theta_{k}\\ \beta_{k} \end{array}\right]+v_{k}$$

## 4. IMMPDA-MDCM Algorithm

The IMM algorithm can estimate the state of a dynamic system with several different models that switch from one to another, and finally get a mixing output. Various nonlinear filtering algorithms can run in the IMM framework [18]. The tracking error of the single IMMPDA algorithm in clutter is large, and obvious error of peak value may appear in the period of target maneuvering. In this paper, the MDCMKF is embedded in IMMPDA architecture for maneuvering target tracking. Assuming there are *r* models, the target dynamics are modeled in Cartesian coordinates as

(12) $$X_{k+1}^{i}=F_{k}^{i}X_{k}^{i}+G^{i}W_{k}^{i},i=1,\cdots ,r$$

Where $X_{k}^{i}$ is the state of the target at time *k* for *i*th model, $F_{k}^{i}$ is the transition matrix of *i*th model, the *G^i^* is the process noise gain matrix. *W^i^* is the mode-dependent process noise sequences with zero mean and covariance *Q^i^*. The transition probability from model i to model j is $P^{ij}$.

(13) $$P=\left[\begin{array}{cccc} P^{11} & P^{12} & \cdots & P^{1r}\\ P^{21} & P^{22} & \cdots & P^{2r}\\ \vdots & \vdots & \cdots & \vdots \\ P^{r1} & P^{r2} & \cdots & P^{rr} \end{array}\right]$$

### 4.1. MDCM Algorithm

In the system of radar/IR compound tacking, the dynamic target is usually modeled and tracked in the Cartesian coordinates, whereas the measurements are provided in terms of range and angle with respect to the radar and IR sensors in the polar coordinates. The debiased converted measurement (DCM) Kalman filter is a popular technique for target tracking. In the spherical coordinate, the true measurements of radar are azimuth angle $\theta_{m}$, elevation angle $\beta_{m}$ and radial distance $r_{m}$, with noise variance as $\sigma_{\theta }^{2}$, $\sigma_{\beta }^{2}$, $\sigma_{r}^{2}$, respectively. The average true deviation $u_{k}$ and average true covariance $R_{k}$ of converted measurement are described as [19]

(14) $$u_{k}={\left[u_{k}^{x},u_{k}^{y},u_{k}^{z}\right]}^{T}$$

(15) $$R_{k}=\left[\begin{array}{l} R_{k}^{xx}R_{k}^{xy}R_{k}^{xz}\\ R_{k}^{yx}R_{k}^{yy}R_{k}^{yz}\\ R_{k}^{zx}R_{k}^{zy}R_{k}^{zz} \end{array}\right]$$

When measurement in the spherical coordinate is converted to be in Cartesian coordinate, the measurement is modified as

(16) $$Z_{c}=Z_{k}-u_{k}=\left[\begin{array}{l} r_{m}\cos\beta_{m}\cos\theta_{m}\\ r_{m}\cos\beta_{m}\sin\theta_{m}\\ r_{m}\sin\beta_{m} \end{array}\right]-u_{k}$$

The debiased converted measurement equation in Cartesian coordinates can be described as

(17) $$Z_{c}=HX_{k}+v_{k}$$

where $H=\left[\begin{array}{ccccccccc} 1 & 0 & 0 & 0 & 0 & 0 & 0 & 0 & 0\\ 0 & 1 & 0 & 0 & 0 & 0 & 0 & 0 & 0\\ 0 & 0 & 1 & 0 & 0 & 0 & 0 & 0 & 0 \end{array}\right]$.

The covariance of the DCM is a noisy stochastic process with strong correlation to the measurement, the filter update equations are actually coupled and nonlinear, which causes the DCM to lose its unbiasedness. In this paper, a modified DCM (MDCM) filter is derived to suppress this dependence. The MDCM filter can be given as follows

Step 1: Initialization

(18) $${\bar{X}}_{0}=E[X_{0}],P_{0}=E[(X_{0}-{\bar{X}}_{0}){\left(X_{0}-{\bar{X}}_{0}\right)}^{T}]$$

Step 2: Predict the target state

(19) $${\bar{X}}_{k+1|k}=F_{k}{\bar{X}}_{k}$$

(20) $$P_{k+1|k}=F_{k}P_{k}F_{k}^{T}+G_{k}Q_{k}G_{k}^{T}$$

Step 3: First update of the target state

(21) $${\bar{K}}_{k+1}=P_{k+1|k}H_{k}^{T}{\bar{S}}_{k}^{-1}$$

(22) $${\bar{S}}_{k}=H_{k}P_{k+1|k}H_{k}^{T}+R_{k}$$

(23) $${\bar{X}}_{k+1}={\bar{X}}_{k+1|k}+K_{k+1}(Z_{k}-u_{k}-H_{k}{\bar{X}}_{k+1|k})$$

(24) $${\bar{P}}_{k+1}=(I-K_{k+1}H_{k})P_{k+1|k}$$

Step 4: Second update of the target state

Step 4.1: Calculate the error covariance using the first target estimation state $\left(\bar{x},\bar{y},\bar{z}\right)$ and covariance $\left(\sigma_{\bar{x}}^{2},\sigma_{\bar{y}}^{2},\sigma_{\bar{z}}^{2}\right)$.

(25) $$\bar{R}\text{(}k+1\text{)=}E[R|\bar{r},\bar{\theta },\bar{\beta }]=\left[\begin{array}{l} {\bar{R}}_{k+1}^{xx}{\bar{R}}_{k+1}^{xy}{\bar{R}}_{k+1}^{xz}\\ {\bar{R}}_{k+1}^{yx}{\bar{R}}_{k+1}^{yy}{\bar{R}}_{k+1}^{yz}\\ {\bar{R}}_{k+1}^{zx}{\bar{R}}_{k+1}^{zy}{\bar{R}}_{k+1}^{zz} \end{array}\right]$$

where ${\bar{R}}_{k+1}^{xx}$,${\bar{R}}_{k+1}^{yy}$,${\bar{R}}_{k+1}^{zz}$,${\bar{R}}_{k+1}^{xy}$,${\bar{R}}_{k+1}^{xz}$ and ${\bar{R}}_{k+1}^{yz}$ are derived by using the radar and IR measurement error.${\bar{R}}_{k+1}^{xx}=0.25({\bar{r}}^{2}+\delta_{\bar{r}}^{2}+\delta_{r}^{2})(1+{\lambda^{\prime }}_{\theta }{\lambda^{\prime }}_{\bar{\theta }}\cos2\bar{\theta })(1+{\lambda^{\prime }}_{\beta }{\lambda^{\prime }}_{\bar{\beta }}\cos2\bar{\beta })-0.25\lambda_{\theta }^{2}\lambda_{\beta }^{2}({\bar{r}}^{2}+\delta_{\bar{r}}^{2})(1+{\lambda^{\prime }}_{\bar{\theta }}\cos2\bar{\theta })(1+{\lambda^{\prime }}_{\bar{\beta }}\cos2\bar{\beta })$, ${\bar{R}}_{k+1}^{yy}=0.25({\bar{r}}^{2}+\delta_{\bar{r}}^{2}+\delta_{r}^{2})(1-{\lambda^{\prime }}_{\theta }{\lambda^{\prime }}_{\bar{\theta }}\cos2\bar{\theta })(1+{\lambda^{\prime }}_{\beta }{\lambda^{\prime }}_{\bar{\beta }}\cos2\bar{\beta })-0.25\lambda_{\theta }^{2}\lambda_{\beta }^{2}({\bar{r}}^{2}+\delta_{\bar{r}}^{2})(1-{\lambda^{\prime }}_{\bar{\theta }}\cos2\bar{\theta })(1+{\lambda^{\prime }}_{\bar{\beta }}\cos2\bar{\beta })$, ${\bar{R}}_{k+1}^{zz}=0.5({\bar{r}}^{2}+\delta_{\bar{r}}^{2}+\delta_{r}^{2})(1-{\lambda^{\prime }}_{\beta }{\lambda^{\prime }}_{\bar{\beta }}\cos2\bar{\beta })-0.5\lambda_{\beta }^{2}({\bar{r}}^{2}+\delta_{\bar{r}}^{2})(1-{\lambda^{\prime }}_{\bar{\beta }}\cos2\bar{\beta })$, ${\bar{R}}_{k+1}^{xy}=0.25{\lambda^{\prime }}_{\theta }{\lambda^{\prime }}_{\bar{\theta }}({\bar{r}}^{2}+\delta_{\bar{r}}^{2}+\delta_{r}^{2})\sin2\bar{\theta }(1+{\lambda^{\prime }}_{\beta }{\lambda^{\prime }}_{\bar{\beta }}\cos2\bar{\beta })-0.25\lambda_{\theta }^{2}\lambda_{\beta }^{2}{\lambda^{\prime }}_{\bar{\theta }}({\bar{r}}^{2}+\delta_{\bar{r}}^{2})\sin2\bar{\theta }(1+{\lambda^{\prime }}_{\bar{\beta }}\cos2\bar{\beta })$,${\bar{R}}_{k+1}^{xz}=0.5\lambda_{\theta }{\lambda^{\prime }}_{\beta }\lambda_{\bar{\theta }}{\lambda^{\prime }}_{\bar{\beta }}({\bar{r}}^{2}+\delta_{\bar{r}}^{2}+\delta_{r}^{2})\cos\bar{\theta }\sin2\bar{\beta }-0.5\lambda_{\theta }\lambda_{\beta }^{2}\lambda_{\bar{\theta }}{\lambda^{\prime }}_{\bar{\beta }}({\bar{r}}^{2}+\delta_{\bar{r}}^{2})\cos\bar{\theta }\sin2\bar{\beta }$,${\bar{R}}_{k+1}^{yz}=0.5\lambda_{\theta }{\lambda^{\prime }}_{\beta }\lambda_{\bar{\theta }}{\lambda^{\prime }}_{\bar{\beta }}({\bar{r}}^{2}+\delta_{\bar{r}}^{2}+\delta_{r}^{2})\sin\bar{\theta }\sin2\bar{\beta }-0.5\lambda_{\theta }\lambda_{\beta }^{2}\lambda_{\bar{\theta }}{\lambda^{\prime }}_{\bar{\beta }}({\bar{r}}^{2}+\delta_{\bar{r}}^{2})\sin\bar{\theta }\sin2\bar{\beta }$,$\bar{r}=\sqrt{{\bar{x}}^{2}+{\bar{y}}^{2}+{\bar{z}}^{2}}$,$\bar{\theta }=\arctan(\bar{y}/\bar{x})$,$\bar{\beta }=\arctan(\bar{z}/\sqrt{{\bar{x}}^{2}+{\bar{y}}^{2}})$, $\lambda_{\bar{\theta }}=e^{-\delta_{\bar{\theta }}^{2}/2}$, $\lambda_{\bar{\beta }}=e^{-\delta_{\bar{\beta }}^{2}/2}$, ${\lambda^{\prime }}_{\bar{\theta }}=e^{-2\delta_{\bar{\theta }}^{2}}=\lambda_{\bar{\theta }}^{4}$, ${\lambda^{\prime }}_{\bar{\beta }}=e^{-2\delta_{\bar{\beta }}^{2}}=\lambda_{\bar{\beta }}^{4}$, $\delta_{\bar{r}}^{2}$, $\sigma_{\bar{\theta }}^{2}$, $\sigma_{\bar{\beta }}^{2}$ are error covariance of $\bar{r}$, $\bar{\theta }$ and $\bar{\beta }$, respectively.

Step 4.2: Update the target state

(26) $$K_{k+1}=P_{k+1|k}H_{k}^{T}S_{k}^{-1}$$

(27) $$S_{k}=H_{k}P_{k+1|k}H_{k}^{T}+{\bar{R}}_{k}$$

(28) $$X_{k+1}={\bar{X}}_{k+1|k}+K_{k+1}(Z_{k}-u_{k}-H_{k}{\bar{X}}_{k+1|k})$$

(29) $$P_{k+1}=(I-K_{k+1}H_{k})P_{k+1|k}$$

### 4.2. IMMPDA-MDCM Algorithm Principle

One complete cycle of the proposed IMMPDA-MDCMKF comprises four major steps: mixing probabilities calculation, IMMPDA-MDCMKF filtering, model probability update and output mixing. Detailed steps of the proposed algorithm is given as follows

Step 1: Mixing probabilities calculation

(30) $$\mu_{k}^{ij}=p^{ij}\mu_{k}^{i}/C_{k}^{j}i,j=1,....,r$$

where $\mu_{k}^{i}$ is the conditional probability of *i*th model at *k*, $C_{k}^{j}$ is the normalizing constant.

(31) $$C_{k}^{j}\text{= }\sum_{i=1}^{r}p^{ij}\mu_{k}^{i}\;j=1,....,r$$

Step 2: MDCM filter in clutter

Step 2.1: Input interaction. Computing the input state and covariance matrices of *i*th model

(32) $$X_{k}^{oi}=\sum_{i=1}^{r}X_{k}^{i}\mu_{k}^{ij}\;$$

(33) $$P_{k}^{oi}=\sum_{i=1}^{r}\left(P_{k}^{i}+(X_{k}^{i}-X_{k}^{oi})\;{\left(X_{k}^{i}-X_{k}^{oi}\right)}^{T}\right)\mu_{k}^{ij}\;$$

Step 2.2: State and covariance prediction

(34) $$X_{k+1|k}^{i}=F_{k}^{i}X_{k}^{oi}$$

(35) $$P_{k+1|k}^{i}=F_{k}^{i}P_{k}^{oi}{\left(F_{k}^{i}\right)}^{T}+G_{k}^{i}Q_{k}^{i}{\left(G_{k}^{i}\right)}^{T}$$

Step 2.3: Validated measurement judgment. The validation region is

(36) $$d_{k+1}^{i(\ell )}={\left(v_{k+1}^{i(\ell )}\right)}^{T}{\left(S_{k+1}^{i(\ell )}\right)}^{-1}v_{k+1}^{i(\ell )},\;\ell =1,2\cdots n$$

(37) $$d_{k+1}^{1(1)}\leq \gamma |\cdots |d_{k+1}^{i(n)}\leq \gamma$$

where $v_{k+1}^{i(\ell )}$ and $S_{k+1}^{i(\ell )}$ are innovation vector and innovation covariance at *k* + 1 of *i*th model. $v_{k+1}^{i(\ell )}=Z_{dk+1}-H_{k+1}^{i}X_{k+1|k}^{i}$, $Z_{dk+1}$ is a matrix with three rows and *n* columns, each column represents a set of measurements, *n* is the number of the measurements. Equation (37) is the validation equation, γ is the threshold corresponding to the gate probability, which can be obtained from Chi-Square tables for a chosen gate probability [20]. Once the *i*th measurement passes the Chi-Square test in Equation (37), it can be utilized in the rest of the probability data association filter.

Step 2.4: Converted measurement error calculation. Calculating $u_{k+1}$ and $R_{k+1}$using Equations (14) and (15).

Step 2.5: Probabilistic data association for each validated measurement.

(38) $$\beta_{k+1}^{i(l)}=\frac{e_{k+1}^{i(l)}}{b_{k+1}^{i}+\sum_{l=1}^{m}e_{k+1}^{i(x)}},l=1,2,...,m$$

(39) $$\beta_{k+1}^{i(0)}=\frac{b_{k+1}^{i}}{b_{k+1}^{i}+\sum_{l=1}^{m}e_{k+1}^{i(x)}}$$

where *m* is the number of validated measurements and associated with the track. $\beta_{k+1}^{i(l)}$ is the association probability of the *i*th target-originated measurement. $\beta_{k+1}^{i(0)}$ is the association probability of all measurements are not valid. $v_{k+1}^{i(mdcm)(l)}$ is the innovation associated with the *l*th validated measurement,$e_{k+1}^{i(x)}=\exp(-0.5{\left(v_{k+1}^{i(mdcm)(l)}\right)}^{T}{\left(S_{k+1}^{i}\right)}^{-1}v_{k+1}^{i(mdcm)(l)})$, $v_{k+1}^{i(mdcm)(l)}=Z_{k+1}^{l}-u_{k+1}-H_{k+1}^{i}X_{k+1|k}^{i}$ $b_{k+1}^{i}=C{\left(2\pi \right)}^{m/2}{\left|S_{k+1}^{i}\right|}^{1/2}(1-P_{D}P_{G})/P_{D}$. *P_D_* and *P_G_* are the target detection probability and the gate probability, respectively.

Step 2.6: First update of the target state and covariance

Using the combined innovation to substitute the clutter-free innovation, and calculate the gain matrix, state and covariance updating matrix.

(40) $${\hat{K}}_{k+1}^{i}=P_{k+1|k}^{i}{\left(H_{k+1}^{i}\right)}^{T}{\left({\hat{S}}_{k+1}^{i}\right)}^{-1}$$

(41) $${\hat{S}}_{k+1}^{i}=H_{k+1}^{i}P_{k+1|k}^{i}{\left(H_{k+1}^{i}\right)}^{T}+R_{k+1}$$

(42) $${\hat{X}}_{k+1}^{i}=X_{k+1|k}^{i}+{\hat{K}}_{k+1}^{i}{\tilde{v}}_{k+1}^{i}$$

(43) $${\tilde{v}}_{k+1}^{i}=\sum_{l=1}^{m}\beta_{k+1}^{i(l)}v_{k+1}^{i(mdcm)(l)}$$

(44) $${\hat{P}}_{k+1}^{i}=P_{k+1|k}^{i}-(1-\beta_{k+1}^{i(0)}){\hat{K}}_{k+1}^{i}{\hat{S}}_{k+1}^{i}{\left({\hat{K}}_{k+1}^{i}\right)}^{T}+{\hat{\tilde{P}}}_{k+1}^{i}$$

(45) $${\hat{\tilde{P}}}_{k+1}^{i}={\hat{K}}_{k+1}^{i}(\sum_{l=1}^{m}\beta_{k+1}^{i(l)}v_{k+1}^{i(mdcm)(l)}{\left(v_{k+1}^{i(mdcm)(l)}\right)}^{T}-{\tilde{v}}_{k+1}^{i}{\left({\tilde{v}}_{k+1}^{i}\right)}^{T}){\left({\hat{K}}_{k+1}^{i}\right)}^{T}$$

Step 2.7: Second update of the target state and covariance. Calculating ${\bar{R}}_{k+1}$ using Equation (25).

(46) $$K_{k+1}^{i}=P_{k+1|k}^{i}{\left(H_{k+1}^{i}\right)}^{T}{\left(S_{k+1}^{i}\right)}^{-1}$$

(47) $$S_{k+1}^{i}=H_{k+1}^{i}P_{k+1|k}^{i}{\left(H_{k+1}^{i}\right)}^{T}+{\bar{R}}_{k+1}$$

(48) $$X_{k+1}^{i}=X_{k+1|k}^{i}+K_{k+1}^{i}{\tilde{v}}_{k+1}^{i}$$

(49) $${\tilde{v}}_{k+1}^{i}=\sum_{l=1}^{m}\beta_{k+1}^{i(l)}v_{k+1}^{i(mdcm)(l)}$$

(50) $$P_{k+1}^{i}=P_{k+1|k}^{i}-(1-\beta_{k+1}^{i(0)})K_{k+1}^{i}S_{k+1}^{i}{\left(K_{k+1}^{i}\right)}^{T}+{\tilde{P}}_{k+1}^{i}$$

(51) $${\tilde{P}}_{k+1}^{i}=K_{k+1}^{i}(\sum_{l=1}^{m}\beta_{k+1}^{i(l)}v_{k+1}^{i(mdcm)(l)}{\left(v_{k+1}^{i(mdcm)(l)}\right)}^{T}-{\tilde{v}}_{k+1}^{i}{\left({\tilde{v}}_{k+1}^{i}\right)}^{T}){\left(K_{k+1}^{i}\right)}^{T}$$

Step 3: Model probability update

(52) $$\mu_{k+1}^{i}=\frac{1}{\bar{c}}\Lambda_{k+1}^{i}c_{i},\bar{c}=\sum_{i=1}^{r}\Lambda_{k+1}^{i}c_{i}$$

where $\Lambda_{k+1}^{i}$ is the likelihood function of *i*th model in IMMPDA-MDCM.

(53) $$\Lambda_{k+1}^{i}=\frac{P_{D}V_{k+1}^{i(-m+1)}}{m}[b_{k+1}^{i}+\sum_{l=1}^{m}e_{k+1}^{i(l)}]$$

(54) $$V_{k+1}^{i}=c_{n_{z}}\gamma^{n_{z}/2}{\left|S_{k+1}^{i}\right|}^{1/2}$$

(55) $$c_{n_{z}}=\{\begin{array}{c} \frac{\pi^{n_{z}/2}}{(n_{z}/2)!},\;n_{z}=2,4,...\\ \frac{2\left(\frac{n_{z}+1}{2}\right)!\pi^{(n_{z}-1)/2}}{(n_{z}+1)!},n_{z}=1,3,... \end{array}$$

Step 4: Output Mixing.

The final target state estimation and covariance matrix are combined from all of the models

(56) $$X_{k+1}=\sum_{i=1}^{r}X_{k+1}^{i}\mu_{k+1}^{i}$$

(57) $$P_{k+1}=\sum_{i=1}^{r}(P_{k+1}^{i}+(X_{k+1}^{i}-X_{k+1}){\left(X_{k+1}^{i}-X_{k+1}\right)}^{T})\mu_{k+1}^{i}$$

## 5. Simulation and Results

**Figure 3:**
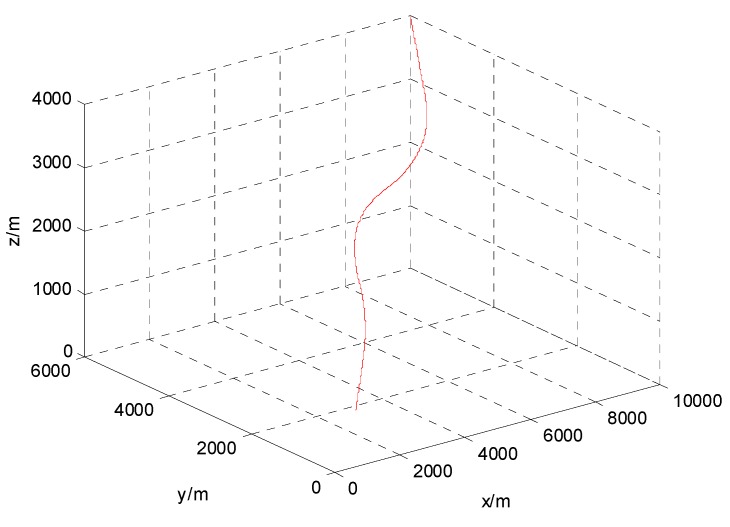
Trajectory of target.

The following example of tracking a highly maneuvering unmanned aerial vehicle is considered. The scenario of a highly maneuvering airborne target tracking is defined as follows: the sampling rate is T=0.1 s, the target makes five accelerating maneuver with linear segments connecting it. The initial position of the target is (10,000, 6000, 4000) m, and the velocity is (−300, −300, −100) m/s. In the first period of 1–5 s, it flies linearly by constant velocity. From 6–10 s, it makes an accelerating maneuver with (20, 50, 0) m/s^2^. From 11–15 s, it flies with (5, 25, 0) m/s^2^. From 16–20 s, it flies with (5, −25, 0) m/s^2^. From 21–25 s, it flies with (−25, −50, 0) m/s^2^. From 26–30 s, it flies with (0, 25, 0) m/s^2^. At last, it flies linearly from 31–35 s by constant velocity. The trajectory of target is shown in Figure 3.

In this paper, the target dynamics are modeled in Cartesian coordinates as Equation (12). The constant velocity (CV) model and Singer model are put into the IMM frame, The CV model is used to describe the basic motion of the target, the Singer model is used to describe target maneuver. 

The state transition matrix and noise gain matrix of CV model are defined as

$$F_{k}^{CV}=\left[\begin{array}{ccccccccc} 1 & 0 & 0 & T & 0 & 0 & 0 & 0 & 0\\ 0 & 1 & 0 & 0 & T & 0 & 0 & 0 & 0\\ 0 & 0 & 1 & 0 & 0 & T & 0 & 0 & 0\\ 0 & 0 & 0 & 1 & 0 & 0 & 0 & 0 & 0\\ 0 & 0 & 0 & 0 & 1 & 0 & 0 & 0 & 0\\ 0 & 0 & 0 & 0 & 0 & 1 & 0 & 0 & 0\\ 0 & 0 & 0 & 0 & 0 & 0 & 0 & 0 & 0\\ 0 & 0 & 0 & 0 & 0 & 0 & 0 & 0 & 0\\ 0 & 0 & 0 & 0 & 0 & 0 & 0 & 0 & 0 \end{array}\right],G_{k}^{CV}=\left[\begin{array}{ccc} T^{2}/2 & 0 & 0\\ 0 & T^{2}/2 & 0\\ 0 & 0 & T^{2}/2\\ T & 0 & 0\\ 0 & T & 0\\ 0 & 0 & T\\ 0 & 0 & 0\\ 0 & 0 & 0\\ 0 & 0 & 0 \end{array}\right]$$

The state transition matrix and noise gain matrix of Singer model are defined as reference [21].

$$F_{k}^{S}=\left[\begin{array}{ccccccccc} 1 & 0 & 0 & T & 0 & 0 & \phi_{17} & 0 & 0\\ 0 & 1 & 0 & 0 & T & 0 & 0 & \phi_{28} & 0\\ 0 & 0 & 1 & 0 & 0 & T & 0 & 0 & \phi_{39}\\ 0 & 0 & 0 & 1 & 0 & 0 & \phi_{47} & 0 & 0\\ 0 & 0 & 0 & 0 & 1 & 0 & 0 & \phi_{58} & 0\\ 0 & 0 & 0 & 0 & 0 & 1 & 0 & 0 & \phi_{69}\\ 0 & 0 & 0 & 0 & 0 & 0 & e^{-\alpha_{x}T} & 0 & 0\\ 0 & 0 & 0 & 0 & 0 & 0 & 0 & e^{-\alpha_{y}T} & 0\\ 0 & 0 & 0 & 0 & 0 & 0 & 0 & 0 & e^{-\alpha_{z}T} \end{array}\right],G_{k}^{S}=\left[\begin{array}{c} G_{1}\\ G_{2}\\ G_{3} \end{array}\right]$$

where, $\phi_{17}=(\alpha_{x}T-1+e^{-\alpha_{x}T})/\alpha_{x}^{2}$, $\phi_{28}=(\alpha_{y}T-1+e^{-\alpha_{y}T})/\alpha_{y}^{2}$, $\phi_{39}=(\alpha_{z}T-1+e^{-\alpha_{z}T})/\alpha_{z}^{2}$, $\phi_{47}=(1-e^{-\alpha_{x}T})/\alpha_{x}$, $\phi_{58}=(1-e^{-\alpha_{y}T})/\alpha_{y}$, $\phi_{69}=(1-e^{-\alpha_{z}T})/\alpha_{z}$, $G_{\text{1}}=diag\left(\left[\begin{array}{c} \sigma_{ax}\left[1-\alpha_{x}T-e^{-\alpha_{x}T}+(T^{2}\alpha_{x}^{2}/2)\right]/\alpha_{x}^{3}\\ \sigma_{ay}\left[1-\alpha_{y}T-e^{-\alpha_{y}T}+(T^{2}\alpha_{y}^{2}/2)\right]/\alpha_{y}^{3}\\ \sigma_{az}\left[1-\alpha_{z}T-e^{-\alpha_{z}T}+(T^{2}\alpha_{z}^{2}/2)\right]/\alpha_{z}^{3} \end{array}\right]\right)$, $G_{\text{2}}=diag\left(\left[\begin{array}{c} \sigma_{ax}(\alpha_{x}T+e^{-\alpha_{x}T}-1)/\alpha_{x}^{2}\\ \sigma_{ay}(\alpha T+e^{-\alpha_{y}T}-1)/\alpha_{y}^{2}\\ \sigma_{az}(\alpha T+e^{-\alpha_{z}T}-1)/\alpha_{z}^{2} \end{array}\right]\right)$, $G_{\text{3}}=diag\left(\left[\begin{array}{c} \sigma_{ax}(1-e^{-\alpha_{x}T})/\alpha_{x}\\ \sigma_{ay}(1-e^{-\alpha_{y}T})/\alpha_{y}\\ \sigma_{az}(1-e^{-\alpha_{z}T})/\alpha_{z} \end{array}\right]\right)$. $\alpha_{x}=\alpha_{y}=\alpha_{z}=\alpha$, α=0.1 is the reciprocal of the manoeuver time constant. $\sigma_{\text{a}x}$, $\sigma_{ay}$, $\sigma_{az}$ are standard deviation of maneuver acceleration in x, y and z direction.

(58) $$\sigma_{\left(ax,ay,az\right)}^{2}=a_{(x,y,z)\max}^{2}(1+4p_{\max}-p_{0})/3$$

$a_{(x,y,z)\max}$($a_{x\max}\text{=}25$, $a_{y\max}\text{=}50$, $a_{z\max}\text{=}0$) is maximum acceleration of target, $p_{\max}=0.5$ is the maximum probability of acceleration or deceleration, $p_{0}=0.5$ is the probability of without acceleration. 

The measuring period of radar $T_{R}=\text{0.01 s}$, the measuring period of IR $T_{I}=\text{0.005 s}$. The range measurement variance of radar is $\sigma_{r_{R}}=100$, the azimuth and elevation angle measurement variance of radar are $\sigma_{\theta_{R}}=\sigma_{\beta_{R}}=0.02$. The azimuth and elevation angle measurement variance of IR sensor are$\sigma_{\theta_{I}}=\sigma_{\beta_{I}}=0.002$. The initial prior probability of the two models are $\mu_{1}=0.5$ and $\mu_{2}=0.5$. That is to say, CV model has the same chance to be selected in the initialization. γ=16,λ=4e−5, $P_{G}=0.997$, $P_{D}=1$. The variances of the process noise of two models are $Q^{CV}=0.05625\cdot I_{3}\cdot {\text{m/s}}^{\text{2}}$, $Q^{S}=3\cdot I_{3}\cdot {\text{m/s}}^{\text{2}}$. $I_{3}$ is the identity matrix of three dimensions. Considering the different process noise level, the transition probability of the system model is chosen as

$$p=\left[\begin{array}{cc} 0.99 & 0.01\\ 0.01 & 0.99 \end{array}\right]$$

**Figure 4:**
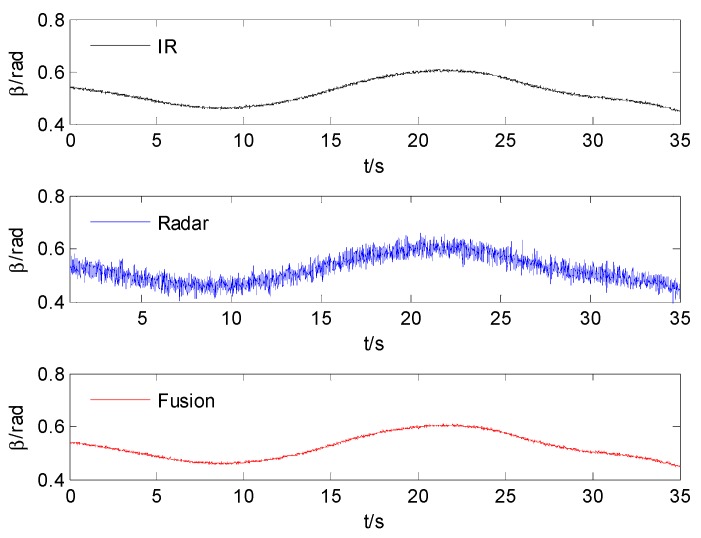
The comparison of azimuth.

**Figure 5:**
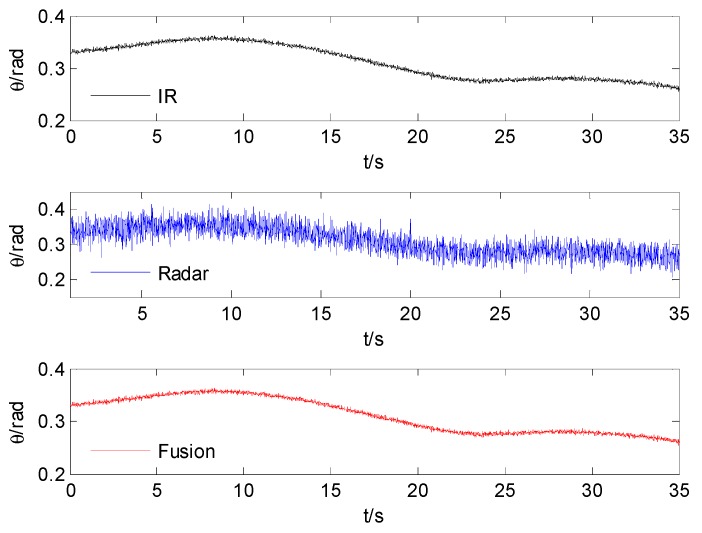
The comparison of elevation.

The azimuth and elevation comparison after fusion are shown in Figure 4 and Figure 5. The comparison standard deviation of azimuth and elevation after time alignment and fusion are shown in Table 1. 

**Table 1:** Standard deviation comparison.

	Before Time Alignment		After Time Alignment		Fusion
	Radar	IR	Radar	IR	
Azimuth (rad)	0.02	0.002	0.02	0.0026	0.0026
Elevation (rad)	0.02	0.002	0.02	0.0026	0.0026

**Figure 6:**
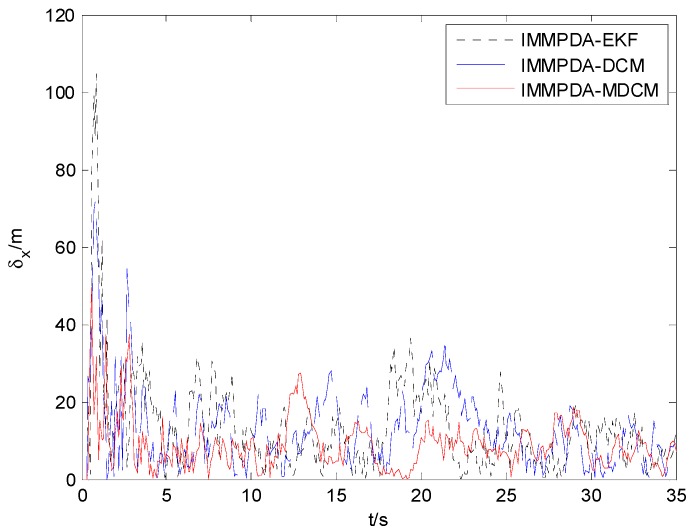
The comparison of position error in x direction.

**Figure 7:**
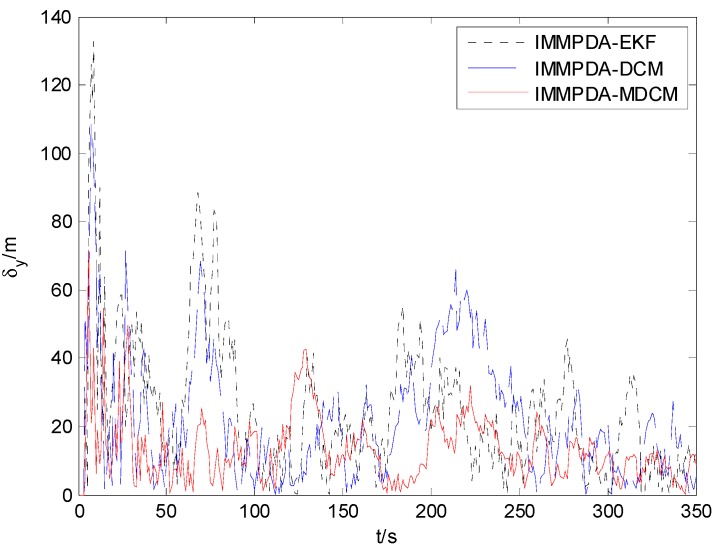
The comparison of position error in y direction.

**Figure 8:**
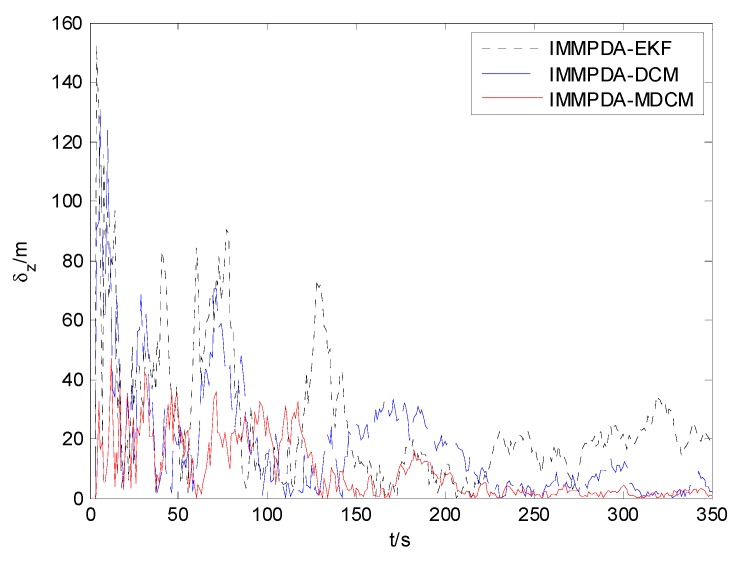
The comparison of position error in z direction.

The tracking performances of proposed IMMPDA-MDCM algorithm, IMMPDA-DCM and IMMPDA-EKF are compared via 100 Monte Carlo simulations. All the algorithms are implemented using a personal computer (Windows 7 2009, Intel Core2 Duo CPU, 2.94 GHz, 4.0 GB of RAM, and MATLAB R2012a programming environment). The results of the root mean square error (RMSE) and runtime test of the target’s position for the three algorithms are shown in Table 2. Figure 6, Figure 7 and Figure 8 show the obtained position estimation error of three algorithms in *x*, *y*, and *z* direction, respectively. 

**Table 2:** The RMSE and runtime comparison of three different algorithms.

	*X* (m)	*Y* (m)	*Z* (m)	*t* (s)
IMMPDA-EKF	13.368	24.379	25.476	4.735
IMMPDA-DCM	12.911	21.803	16.965	0.767
IMMPDA-MDCM	8.891	13.205	12.203	1.396

The proposed algorithm can calculate the statistic characteristics of converted measurement errors and make the covariance to be much less noisy. As can be seen in Figure 6, Figure 7 and Figure 8 , the proposed IMMPDA-MDCM algorithm has the highest tracking precision than IMMPDA-DCM and IMMPDA-EKF algorithm, which is consistent with the results in Table 2. The total position tracking error of the proposed algorithm is reduced by 34.22% and 46.81% compared to IMMPDA-DCM and IMMPDA-EKF, respectively. In the simulation, the two update of IMMPDA-MDCM will increase thecalculation time; and the computational cost of IMMPDA-MDCM is longer than IMMPDA-DCM, but shorter than IMMPDA-EKF.

## 6. Conclusions

In this paper, an interacting multiple model probability data association algorithm based on modified debiased converted measurement filter (IMMPDA-MDCM) is proposed, which is capable of adaptively tracking the maneuvering airborne target. The polar measurements of radar and IR measurements are time aligned, fused and converted to Cartesian coordinates before they are applied to IMMPDA-MDCM algorithm. In the IMMPDA-MDCM algorithm, the covariance of the converted measurement is recalculated using the estimated target position information. Therefore, the proposed algorithm can reduce the effect of measurement noise on the covariance effectively. By abandoning the extended Kalman filter framework and using MDCM filter in the proposed algorithm, the linearization errors of the measurement model are avoided, and the good tracking precision is achieved with decreasing the computational complexity. Monte Carlo simulation results verify that the proposed algorithm outperforms IMMPDA-DCM and IMMPDA-EKF in terms of filtering unbiasedness and precision. The proposed algorithm is an effective algorithm for maneuvering target tracking in clutter, which can increase warfare airplane’s concealment and survival capacity.
